# Preliminary analysis of in utero low-level arsenic exposure and fetal growth using biometric measurements extracted from fetal ultrasound reports

**DOI:** 10.1186/1476-069X-14-12

**Published:** 2015-03-30

**Authors:** Matthew A Davis, John Higgins, Zhigang Li, Diane Gilbert-Diamond, Emily R Baker, Amar Das, Margaret R Karagas

**Affiliations:** Children’s Environmental Health and Disease Prevention Research Center at Dartmouth, Hanover, NH USA; Institute for Quantitative Biomedical Sciences Graduate Program, Dartmouth College, Hanover, NH USA; University of Michigan School of Nursing, Ann Arbor, MI USA; Department of Epidemiology, Geisel School of Medicine at Dartmouth, Hanover, NH USA; Collaboratory for Healthcare and Biomedical Informatics, Geisel School of Medicine at Dartmouth, Hanover, NH USA; Department of Biostatistics, Geisel School of Medicine at Dartmouth, Hanover, NH USA; Dartmouth Hitchcock Medical Center, Lebanon, NH, USA; Department of Epidemiology, Geisel School of Medicine at Dartmouth, One Medical Center Drive, 7927 Rubin Building, 03756 Lebanon, NH USA

**Keywords:** Arsenic, Prenatal exposure, Fetal development

## Abstract

**Background:**

Early life exposure to arsenic is associated with decreased birth weight in highly exposed populations but little is known about effects of low-level arsenic exposure on growth in utero.

**Methods:**

Using a sample of 272 pregnancies from New Hampshire we obtained biometric measurements directly from fetal ultrasound reports commonly found in electronic medical records. We used information extraction methods to develop and validate an automated approach for mining biometric measurements from the text of clinical reports. As a preliminary analysis, we examined associations between in utero low-level arsenic exposure (as measured by maternal urinary arsenic concentration) and fetal growth measures (converted to Z-scores based on reference populations for estimated fetal weight, head, and other body measures) at approximately 18 weeks of gestation.

**Results:**

In a preliminary cross-sectional analysis of 223 out of 272 pregnancies, maternal urinary arsenic concentration (excluding arsenobetaine) was associated with a reduction in head circumference Z-score (Spearman correlation coefficient, r_s_ = -0.08, p-value = 0.21) and a stronger association was observed among female fetuses at approximately 18 weeks of gestation (r_s_ = - 0.21, p-value < 0.05). Although, associations were attenuated in adjusted analyses — among female fetuses a 1 μg/L increase in maternal urinary arsenic concentration was associated with a decrease of 0.047 (95% CI: -0.115, 0.021) in head circumference and 0.072 (95% CI: -0.151, 0.007) decrease in biparietal head diameter Z-score.

**Conclusions:**

Our study demonstrates that useful data can be extracted directly from electronic medical records for epidemiologic research. We also found evidence that exposure to low-level arsenic may be associated with reduced head circumference in a sex dependent manner that warrants further investigation.

**Electronic supplementary material:**

The online version of this article (doi:10.1186/1476-069X-14-12) contains supplementary material, which is available to authorized users.

## Background

Arsenic is a metalloid found throughout the world and human exposure through ingestion of contaminated water and food has been associated with a wide array of health effects [1]. More recently, attention has turned to examining the effects of early life exposure to arsenic on both early childhood and adult health outcomes [2, 3]. In highly exposed populations, early life exposure to arsenic has been associated with adverse birth outcomes such as increased risk of infection and diarrheal disease and higher infant mortality [4–10] as well as childhood neurobehavioral effects [3, 11–15]. Furthermore, a variety of health effects have been observed into adulthood resulting from early life exposure including higher mortality rates from bronchiectasis and cancers [16–19], cardiovascular disease [20], and all cause mortality [17].

The developing fetus is particularly vulnerable to stress and environmental toxicants [2, 21–23]. Inorganic arsenic and its metabolites readily cross the placental barrier and appear in fetal tissue [24, 25]. In animal studies, arsenic has been shown to have teratogenic and embryologic toxic effects [26] and research has uncovered effects on gene expression [27]. While previous studies have found effects on birth size [7] and growth into childhood [28] much less is known about effects on growth in the earliest stages of life [29]. To our knowledge only one study conducted in a highly exposed population has examined arsenic’s effects on intrauterine growth and found growth impairment in a sex-dependent manner [10]. Due to differences in sociodemographic characteristics and other factors that influence pregnancy, findings from highly exposed populations are not necessarily generalizable to other parts of the world with relatively lower levels of exposure such as the US. One previous study from Bangladesh found that effects on human growth may occur at the lower end of the dose response curve (less than 100 ug/L as measured in urine) suggesting populations experiencing lower levels of exposure may be affected [7].

Recently investigators are turning to clinical narratives and free-text reports found in electronic medical records (EMRs) to extract critical health information that is not captured in a structured, coded format [30]. In particular, information extraction techniques are used to obtain information from unstructured or semi-structured text. Since the late 1950s obstetrical ultrasound has been used to inform prenatal care among obstetricians and midwives. Clinically, the fetal ultrasound examination is a diagnostic procedure used for evaluating fetal weight and growth, estimating gestational age, and for examining organ function. At approximately 18 weeks of gestation the fetus is large enough to obtain accurate assessments of growth and development. Because of the highly structured (and consistent) format of the text generally found in radiological and imaging reports, fetal ultrasound reports are excellent candidates for automated information extraction techniques.

Therefore in a sample of 272 pregnancies from New Hampshire we sought to extract biometric measurements directly from fetal ultrasound reports found in EMRs. To demonstrate the utility of automated techniques for epidemiologic study, we also performed a preliminary analysis to examine the relationship between in utero exposure to low levels of arsenic and fetal growth.

## Methods

### Study population

The New Hampshire Birth Cohort Study (NHBCS) is an ongoing prospective study that began in 2009 and currently includes over 1,000 women from New Hampshire, between the ages 18 and 45 years, with a singleton pregnancy and who report having a private well as their primary home water source. During enrollment at a study clinic (typically at 24-28 weeks of gestation), study participants provide a spot urine sample and complete a prenatal questionnaire that collects information about their health and pregnancy. Among a variety of environmental exposures, the NHBCS is particularly focused on health outcomes associated with low level arsenic exposure, which is common among those in New Hampshire who consume water from unregulated well water. In this study population well water total arsenic concentration was less than 10 μg/L (ranged from 0.0 to 9.3 μg/L) for all study participants.

**Figure 1:**
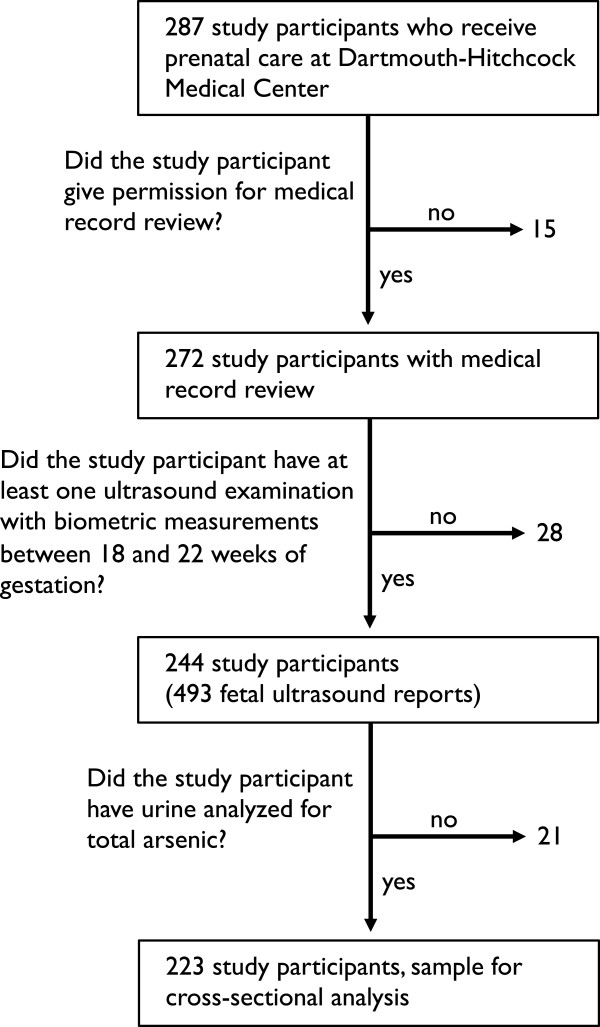
**Flow diagram of study participant inclusion for extraction of fetal biometric measurements from ultrasound reports.**

Study participants are asked for permission to review medical record review to gather additional information about the pregnancy and birth outcomes. For the 272 study participants who received medical care at Dartmouth Hitchcock Medical Center in Lebanon, New Hampshire, and gave permission for the medical record review, we identified 244 study participants who had at least one fetal ultrasound examination between 18 and 22 weeks of gestation, the recommended initial morphologic ultrasound exam to assess development, that included fetal growth measurements (Figure 1) [31].

The study protocol for the NHBCS was approved by the Committee for the Protection of Human Subjects at Dartmouth College. All study participants provided written informed consent.

### Fetal biometric measurements from clinical ultrasound reports

It is common for pregnant women in the US to undergo at least one fetal ultrasound examination between 18 and 22 weeks of gestation. Morphologic fetal assessment during an ultrasound examination records biometric measurements including head circumference (HC), biparietal diameter (BPD), abdominal circumference (AC), and femur length (FL). At Dartmouth Hitchcock Medical Center, BPD is measured from outer to inner portions of the calvarial wall of the skull, and other measurements including HC, FL, and AC are all measured from outer to outer portions of the respective anatomy by trained sonographers. From HC, AC and FL biometric measurements, we estimated fetal weight (EFW) using the Hadlock III equation: log_10_EFW = 1.326 – (0.00326 × AC × FL) + (0.0107 × HC) + (0.0438 × AC) + (0.158 × FL) [32]. Fetal ultrasound examinations at Dartmouth Hitchcock Medical Center are performed on either a General Electric Voluson (General Electric Healthcare Corporate Headquarters, Little Chalfont, Buckinghamshire, UK) or a Phillips iU22 (Philips Healthcare, Andover, Massachusetts, USA).

Working with the Collaboratory of Healthcare and Biomedical Informatics, we queried the EMRs of NHBCS participants who received prenatal care at Dartmouth Hitchcock Medical Center and who gave permission to have their medical records reviewed for current procedural terminology (CPT) codes for ultrasound examinations including: 59000, 76801, 76805, 76811, 76812, 76815, 76816, 76817, 76819, and 76820 by cross-linking study participants’ identification number with their medical record number. After removing ultrasound reports that did not include biometric measurements and mothers who did not have at least one ultrasound exam between 18 and 22 weeks of gestation, we identified a total of 493 ultrasound reports (Figure 1 and Table 1).

**Table 1:** **Ultrasound reports according to current procedural terminology code.**

CPT code	Description	n (%)
76816	Ultrasound pregnant uterus, follow-up evaluation	233 (47.3)
76805	Ultrasound pregnant uterus, fetal and maternal evaluation	210 (42.6)
76811	Ultrasound pregnant uterus, fetal and maternal targeted evaluation	38 (7.7)
59000	Ultrasound pregnant uterus, guided amniocentesis	6 (1.2)
76815	Ultrasound pregnant uterus, limited evaluation	3 (0.6)
76812	Ultrasound pregnant uterus, targeted evaluation multiple	2 (0.4)
76801	Ultrasound pregnant uterus, nuchal translucency	1 (0.2)
	Total:	493 (100.0)

Abbreviations: *CPT*, current procedural terminology.

**Figure 2:**
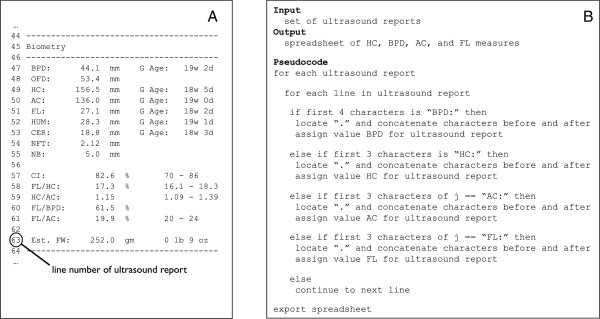
**Example section of ultrasound report that includes fetal biometric measurements (A) and pseudocode used to extract measurements (B).**

While most women receive one fetal ultrasound examination during their pregnancy, women who at higher risk for adverse birth outcomes or those who are experiencing complications may receive repeated fetal ultrasound examinations throughout pregnancy. Among the ultrasound reports we identified, 42.6% of our sample of ultrasound reports were for CPT code 76805 which corresponds to an initial fetal ultrasound examination, “Ultrasound pregnant uterus, fetal and maternal evaluation,” and 47.3% for CPT code 76816 that corresponds to a follow-up ultrasound, “Ultrasound pregnant uterus, follow-up evaluation” (Table 1).An automated technique was developed to extract biometric measurements directly from the text of clinical ultrasound reports obtained from study participants’ EMRs. All ultrasound reports were reposited in a structural query language (SQL) relational database allowing us to assign a collection of fetal ultrasound reports to a specific NHBCS participant. Fetal ultrasound reports obtained from EMRs were in a narrative form consisting of approximately 100 to 150 lines stored as text strings. The narrative was separated into “Patient information,” “Performed by,” “Procedures,” “Indications,” “Fetal evaluation,” “Biometry,” “Gestational age,” “Anatomy,” and “Impression” sections. We focused our attention on the section reporting biometric measurements within each report (Figure 2A).Using information extraction techniques with regular expressions, an automated method to search the text was developed (i.e. each text string in each ultrasound report) to find sections that began with either “BPD:,” “HC:,” “AC:,” or “FL:.” For sections that began with these prefixes the program located the character “.”, concatenated characters before and after to obtain the measurement, and assigned this value to the respective biometric measurement (Figure 2B). After the initial extraction of biometric measurements from all ultrasound reports, text strings were converted to numeric variable formats and merged with other data obtained by the NHBCS.

To assess the accuracy of the automated tool, one member of the study staff of the NHBCS reviewed a random sample of 50 total fetal ultrasound reports. For each report, the staff member manually recorded HC, BPD, AC, and FL and compared the measurements to those our tool extracted. Based on this review, the *recall* (i.e., sensitivity) and *precision* (i.e., positive predictive value) of our tool were both 100% for the set of 200 (four measurements per report) biometric measurements.

### Arsenic exposure assessment

We used maternal urinary arsenic concentrations obtained at approximately 24-28 weeks of gestation as our measure of in utero arsenic exposure. Spot urine samples were stored frozen at -20°C or lower until analysis at the University of Arizona. Samples were analyzed for individual species of arsenic using a combination of high performance liquid chromatography (HPLC) and inductively coupled plasma mass spectrometry (ICPMS) that is able to detect five arsenic species, including arsenate, arsenite, monomethylarsonic acid (MMA), dimethylarsinic acid (DMA), and arsenobetaine [33]. Detection limits for the five individual arsenic species ranged from 0.10 to 0.15 μg/L. The total number of samples below the detection limit were 160 (71.7%), 100 (44.8%), 4 (1.8%), and 0 (0.0%) for arsenate, arsenite, MMA, and DMA respectively. Samples below the detection limit were assigned a value equal to the detection limit divided by 2. Unmetabolized arsenobetaine is believed to be essentially non-toxic, therefore we estimated total arsenic by summing arsenate, arsenite, MMA, and DMA [34, 35].

Metabolism of inorganic arsenic differs among individuals and the relative concentration of urinary arsenic species can used to estimate arsenic methylation capacity [36]. Therefore, we examined maternal arsenic metabolism (as measured by relative urinary arsenic species) as a potential effect modifier of in utero arsenic exposure and fetal growth. To do so, we used the ratio of MMA to inorganic arsenic (the sum of arsenate and arsenite) as a measure of efficiency for the 1^st^ methylation step and the ratio of DMA to MMA for the 2^nd^ methylation step of arsenic metabolism. For both ratios we identified mothers above and below the median to estimate mothers who exhibited higher versus lower arsenic metabolic efficiency.

### Other measures

We examined a variety of factors related to the mother and fetus as potential covariates in our analyses that were collected from a combination of a prenatal questionnaire and medical record review. Maternal factors included age upon study enrollment, pre-pregnancy body mass index (BMI, kg/m^2^), smoking status during pregnancy, and parity and we also examined fetal sex.

Gestational age was used to determine age-specific Z-scores for growth measures and was estimated in two ways in our data. We used the self-reported last menstrual period (LMP) for the 133 study participants whose estimates of gestational age were confirmed with the fetal ultrasound exam. For 90 study participants (26 of which had no known LMP and 64 who’s LMP did not align with dating from fetal ultrasound exam) gestational age was determine by fetal ultrasound.

To account for differences in maternal urinary dilution, Cayman’s Creatinine Assay Kit (Cayman Chemical Company, Ann Arbor, Michigan, USA) was used to determine urinary creatinine.

### Statistical analyses

For each of the 223 mothers with urinary arsenic concentration measured, one fetal ultrasound examination with complete fetal biometric measurements was assigned between the 18 and 22 weeks of gestation. Sixteen out of the 223 women had more than fetal ultrasound with biometric measurements during this window and for these mothers the ultrasound examination closest to the median (18.4 weeks) was assigned.

For all analyses, biometric measurements from ultrasound reports (HC, BPD, AC, and FL) as well as our calculated EFW were converted to age-specific Z-scores. We explored using reported standard equations from a variety of reference populations, and based on visual fit among mothers in the NHBCS (244 pregnancies that had a total of 493 ultrasound examinations), we chose a reference population from England for head and body measures and a reference population from the Netherlands for EFW (Additional file 1: Figure S1) [37–41]. Use of the reference distribution from England also allowed us to compare our results to those of the only other paper in the literature [10]. For EFW, as reported in the literature, separate equations were used to estimate age-specific Z-score before and after 36 weeks of gestation.

We examined relationships between maternal urinary arsenic concentration and fetal growth in several ways. We used Spearman correlation to assess crude relationships, LoWeSS (locally weighted scatterplot smoothing) to visually identify potential non-linear associations, and linear regression to assess associations adjusted for confounding factors. We explored these associations stratified by fetal sex and according to maternal arsenic metabolism. For all models, associations were based on an increase of 1 μg/L of maternal total urinary arsenic concentration. We assessed a variety of maternal and fetal characteristics as potential covariates in our regression models (Table 2). Based on these analyses, covariates that effectively changed (operationally defined as changing the point estimate more than 10% when included in the model) estimates of associations in our cross-sectional analysis were included as covariates for each respective growth outcome. Although it did not effectively change our point estimate, we also included maternal urinary creatinine to account for urinary dilution in our models. For these models maternal age upon study enrollment, maternal BMI, parity, and maternal urinary creatinine were included as continuous variables. All regression models were based on complete case analysis for covariates except for urinary creatinine to which we assigned the 59 mothers with missing values the median creatinine value (67.7 mg/dL). Statistical analyses were conducted using Stata MP version 13.1 (StataCorp, College Station, Texas, USA). We set a p-value for statistical significance to 0.05 (2-sided).

**Table 2:** **Mean Z-score [standard deviation] for fetal biometric measurements at approximately 18 weeks of gestation according to maternal and fetal characteristics**

			Head measures		Body measures	
Variable	n (%)	Estimated fetal weight	Head circumference	Biparietal diameter	Abdominal circumference	Femur length
**Maternal characteristics**						
Age at study enrollment			*	**		*
<30 years	83 (37.2)	0.21 [1.46]	-0.12 [0.82]	-1.19 [1.07]	-0.30 [0.79]	-0.13 [0.85]
≥30 years	140 (62.8)	0.45 [1.46]	0.15 [0.78]	-0.75 [0.92]	-0.29 [0.83]	0.12 [0.80]
Pre-pregnancy BMI, kg/m^2^						
<25.0, normal	111 (49.8)	0.51 [1.48]	0.13 [0.81]	-0.78 [1.08]	-0.19 [0.82]	0.06 [0.82]
≥25.0 to < 30.0, overweight	51 (22.9)	0.41 [1.59]	0.10 [0.90]	-0.96 [0.96]	-0.28 [0.84]	0.64 [0.84]
≥30.0, obese	37 (16.6)	-0.06 [1.20]	-0.22 [0.66]	-1.18 [0.85]	-0.49 [0.79]	-0.14 [0.70]
unknown	24 (10.8)	0.21 [1.42]	-0.03 [0.73]	-1.04 [0.86]	-0.48 [0.76]	0.08 [1.02]
Smoking during pregnancy						
non-smoker	181 (81.2)	0.36 [1.49]	0.05 [0.82]	-0.90 [1.04]	-0.29 [0.84]	0.04 [0.80]
smoker	19 (8.5)	0.41 [1.28]	0.03 [0.74]	-0.96 [0.74]	-0.10 [0.58]	-0.18 [1.00]
unknown	23 (10.3)	0.28 [1.41]	0.06 [0.75]	-1.00 [0.89]	-0.45 [0.78]	0.13 [0.92]
Parity				**		
First live birth	82 (36.8)	0.46 [1.30]	0.14 [0.79]	-0.62 [0.90]	-0.21 [0.71]	0.05 [0.83]
1 or more live births	136 (61.0)	0.28 [1.54]	-0.01 [0.81]	-1.09 [0.99]	-0.35 [0.86]	0.01 [0.82]
unknown	5 (2.2)	0.83 [1.95]	0.23 [0.94]	-1.01 [1.85]	-0.02 [1.17]	0.23 [1.06]
Urinary total arsenic						
below the median	112 (50.2)	0.28 [1.40]	0.07 [0.76]	-0.88 [0.90]	-0.33 [0.83]	-0.04 [0.77]
above the median	111 (49.8)	0.44 [1.52]	0.86 [0.86]	-0.95 [1.09]	-0.26 [0.80]	0.09 [0.88]
**Fetal characteristics**						
Sex			*	**	*	
male	114 (51.1)	0.51 [1.35]	0.20 [0.76]	-0.69 [0.95]	-0.16 [0.76]	0.02 [0.80]
female	101 (45.3)	0.23 [1.53]	-0.10 [0.82]	-1.13 [0.98]	-0.43 [0.84]	0.07 [0.82]
unknown	8 (3.6)	-0.19 [1.91]	-0.27 [0.91]	-1.49 [1.30]	-0.43 [1.07]	-0.38 [1.23]

Abbreviations: *SD*, standard deviation; *BMI*, body mass index.

ANOVA (analysis of variance) used to compare means across categories.

*p-value < 0.05, **p-value < 0.01.

We performed two sensitivity analyses. First, because for 90 out of 223 study participants, gestational age was estimated or informed by the actual ultrasound examination, we examined associations among only those 133 mothers where LMP was used to estimate gestational age. Second, as 16 out of the 223 mothers had more than one ultrasound examination (presumably because they were either higher risk pregnancies or experiencing complications) we re-examined associations only among the 207 mothers who had one ultrasound examination.

## Results

### Study population characteristics

The mean age of women upon study enrollment (between approximately 24 and 28 weeks of gestation) was 31.7 years (range 18.5 to 43.4 years) and over 95% were Non-Hispanic White. The mean pre-pregnancy BMI was 25.9 kg/m^2^ (range 16.6 to 52.5 kg/m^2^) and 19 of the 223 study participants (8.5%) reported smoking during their pregnancy. Eighty two mothers (36.8%) were primiparous and among the multiparous mothers, previous live births ranged from 1 to 6 (Table 2). The median maternal total urinary arsenic concentration (excluding arsenobetaine) obtained between about 24 and 28 weeks of gestation was 3.1 μg/L (IQR: 1.5 to 5.5 μg/L) and ranged from 0.0 to 22.0 μg/L. Approximately half of the fetuses were male (51.1%) (Table 2).

At the time of the 18 to 22 week fetal ultrasound examination, the estimated mean gestational age was 18.4 weeks (range 18.0 to 21.3 weeks). The mean EFW was calculated to be 244.1 g (SD: 36.4 g). The mean HC, BPD, AC, and FL obtained directly from ultrasound examination reports were 156.5 (SD: 8.8), 42.0 (SD: 2.5), 132.8 (SD: 9.3), and 27.9 (SD: 2.1) millimeters respectively. Converted to gestational age specific Z-scores based on reference population, the mean Z-score for EFW, HC, BPD, AC, and FL were 0.36 (SD: 1.5), 0.05 (SD: 0.8), -0.92 (SD: 1.00), -0.29 (SD: 0.82), and 0.03 (SD: 0.83) respectively. Across some maternal characteristics HC, BPD, and AC Z-score differed (Table 2). Most notable, BPD Z-score differed across maternal age categories, parity, and fetal sex (p-value < 0.001 for all). In addition, HC Z-score differed across maternal age category and fetal sex (p-value < 0.05 for both) and FL Z-score by maternal age category (p-value < 0.05).

### In utero arsenic exposure and fetal growth

**Figure 3:**
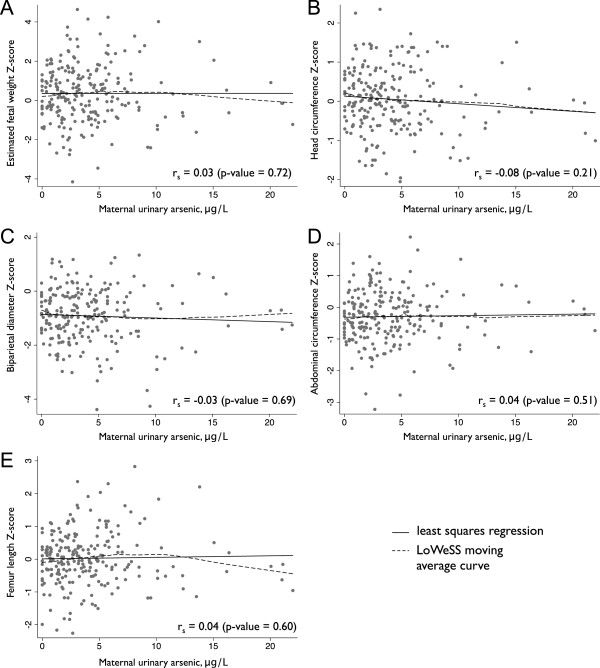
**Unadjusted relationship between maternal total urinary arsenic concentration (excluding arsenobetaine) and estimated fetal weight (A), head circumference (B), biparietal head diameter (C), abdominal circumference (D), and femur length (E) Z-score.** In all panels, r_s_ represents the Spearman correlation coefficient, black lines represent least squares regression, and dashed line represents LoWeSS (locally weighted scatterplot smoothing) moving average fitted curve.

In our aggregate, unadjusted analyses using Spearman correlation we found few apparent associations between in utero arsenic exposure (measured by maternal urinary arsenic concentration) and EFW, HC, BPD, AC, and FL Z-scores (Figure 3). However, a slight inverse association between urinary arsenic concentration and HC Z-score was seen visually (r_s_ = -0.08, p-value = 0.21). In analyses stratified by fetal sex, we found a statistically significant inverse association between HC Z-score and urinary arsenic concentration among female fetuses (r_s_ = - 0.21, p-value < 0.05). This was observed for BPD Z-score as well, although the association did not reach statistical significance (r_s_ = - 0.18, p-value = 0.08).

In our adjusted linear regression models, we found only one statistically significant association between maternal urinary arsenic concentration and fetal growth measures (Table 3). Although not statistically significant our findings are suggestive of inverse associations between arsenic exposure and head growth particularly for female fetuses — effect estimates for HC and BPD Z-score for female fetuses were -0.047 (95% CI: -0.115, 0.021) and -0.072 (95% CI: -0.151, 0.007) respectively.

**Table 3:** **Estimated change in Z-score fetal biometric measurements (95% confidence interval) based on an increase of 1** μ**g/L of maternal urinary arsenic concentration according to fetal sex and maternal arsenic metabolism**

		Head measures		Body measures	
	Estimated fetal weight ^a^	Head circumference ^a^	Biparietal diameter ^a,b^	Abdominal circumference ^a,b^	Femur length ^b,c^
All	-0.003 (-0.061, 0.055)	-0.019 (-0.050, 0.013)	-0.005 (-0.042, 0.033)	0.006 (-0.027, 0.037)	0.001 (-0.031, 0.033)
By fetal sex					
male	0.013 (-0.052, 0.078)	-0.016 (-0.052, 0.021)	0.021 (-0.022, 0.065)	0.011 (-0.025, 0.048)	0.014 (-0.023, 0.051)
female	-0.041 (-0.171, 0.089)	-0.047 (-0.115, 0.021)	-0.072 (-0.151, 0.007)	-0.014 (-0.085, 0.057)	-0.016 (-0.085, 0.053)
By arsenic metabolism (ratio of urinary arsenic metabolites)					
First methylation step (MMA/iAs)					
below median	-0.025 (-0.107, 0.056)	-0.023 (-0.077, 0.017)	-0.007 (-0.066, 0.052)	-0.009 (-0.056, 0.038)	-0.003 (-0.047, 0.041)
above median	0.023 (-0.066, 0.111)	-0.005 (-0.050, 0.040)	0.003 (-0.049, 0.056)	0.021 (-0.027, 0.069)	0.008 (-0.040, 0.057)
Second methylation step (DMA/MMA)					
below median	-0.023 (-0.166, 0.121)	-0.057 (-0.137, 0.023)	-0.010 (-0.193, -0.006)*	-0.019 (-0.098, 0.061)	0.018 (-0.061, 0.098)
above median	0.012 (-0.058, 0.082)	-0.007 (-0.044, 0.029)	0.027 (-0.014, 0.069)	0.021 (-0.017, 0.060)	0.001 (-0.036, 0.038)

Abbreviations: CI, confidence interval; iAs, inorganic arsenic; MMA, monomethylarsonic acid; DMA, dimethylarsinic acid.

*p-value < 0.05.

All models adjusted for maternal pre-pregnancy BMI (kg/m^2^), parity (number), and maternal urinary creatinine (mg/L).

^a^further adjusted for fetal sex.

^b^further adjusted for maternal age (years).

^c^further adjusted for maternal smoking.

Examined by relative maternal arsenic metabolism (ratios of the urinary arsenic metabolites), we found an inverse association between BPD Z-score and urinary arsenic concentration among mothers below the median for DMA/MMA ratio; a 1 μg/L increase in urinary arsenic concentration was associated with a 0.010 decrease in BPD Z-score (95% CI: -0.193, -0.006). We also observed inverse associations were for other growth measures (i.e., with effect estimates ranging from -0.003 to -0.025 for mothers below the median for first step arsenic metabolism), but these could have been due to chance (Table 3).

In our sensitivity analyses restricted to the 133 mothers with known and reliable estimates of gestational age from LMP, the Spearman correlation coefficient between maternal urinary arsenic concentration and HC Z-score was -0.05, p-value = 0.57. Among just female fetuses the Spearman correlation coefficient between urinary arsenic concentration and HC Z-score was -0.09, p-value = 0.54. In our analyses where we excluded the 16 mothers with multiple ultrasound examinations, associations were very similar overall. However, the inverse association between BPD Z-score and urinary arsenic concentration among female fetuses became statistically significant (a 1 μg/L increase in urinary arsenic concentration was associated with a 0.089 (95% CI: 0.170, 0.008) decrease in BPD Z-score).

## Discussion

Prior studies have performed information extraction from pathology and other types of radiography reports; however, our study is among the first to demonstrate the utility of information extraction techniques to accurately obtain fetal biometric measurements from EMRs as assessed during prenatal visits. Our work clearly demonstrates how additional important health information (data not originally collected by the study) can be obtained and used for epidemiologic study.

Validated and automated techniques that can be applied to EMR data have the distinct advantage of being able to collect data more efficiently than manual review. This may be particularly of interest to epidemiologists interested in studying rare outcomes. In our study we validated a tool using data from only one US medical institution. Building upon our approach, future work can further develop and adapt a more universal tool to extract biometric measurements from a variety of different structured fetal ultrasound reports (i.e., fetal ultrasound reports that may be structured differently).

In addition to demonstrating one method of extracting data from EMRs, we also found that exposure to low levels arsenic in the US population may be associated with reduced head growth (measured as HC and BPD) among female fetuses albeit with limited statistical precision. Previous studies have used biometric measurements manually extracted from fetal ultrasound reports to examine the impacts of environmental exposures. For instance, reduced BPD and HC have been related to exposure to aromatic hydrocarbons [22, 23] and maternal factors such as both passive and active exposure to smoke [42–44] and maternal occupational exposure to air pollutants related to impaired fetal most growth measures [45]. The only prior study of arsenic exposure and intrauterine growth to our knowledge was in highly exposed population and found fetal growth effects in sex-specific manner – arsenic exposure was weakly associated with reduced EFW in males [10].

In our relatively small sample we found suggestive inverse associations with in utero arsenic exposure (measured by maternal urinary arsenic concentration) and HC Z-score (r_s_ = -0.08, p-value = 0.21). Future work in larger study populations are required to make more conclusive findings. Similar to prior work, we also found associations between in utero arsenic exposure and fetal growth in sex-dependent manner. Most notably, we found an inverse association between in utero exposure to arsenic and head growth (both HC and BPD) among female fetuses. This is a potentially important finding and suggests that maternal exposure to low-level arsenic may be directly related to changes in fetal growth.

While the long-term health effects of reduced in utero growth remain unclear, there is emerging evidence that intrauterine growth may be related to birth outcomes and health later in life [46]. For instance, early fetal growth has been associated with two important birth outcomes: birth weight and gestational age of delivery [47]. Early life head growth in particular has been associated with a number of different early childhood health outcomes including motor, cognitive, and other neurodevelopmental effects [48–51]. Furthermore, a recent large study has found growth during the first trimester (as measured by crown to rump fetal length) to be associated with cardiovascular outcomes later in childhood [52]. Collectively, mounting evidence does suggest that early growth is an important predictor of a variety of downstream health effects.

In regards to our preliminary analyses of in utero arsenic exposure and fetal growth, there are a number of important limitations of our study that must be acknowledged. First, our cross-sectional analysis consisted of only 223 mothers who received prenatal care at a medical institution. While even among this small sample we did find suggestive associations, our study was statistically underpowered and therefore our findings are not conclusive (our study was powered at 0.32 to detect an association of -0.1 and 0.85 to detect an association of -0.2). Moreover, the generalizability of our findings is limited to mothers who receive prenatal medical care (effects may differ among mothers who do not receive prenatal care as in other parts of the world). Second, standardized equations to calculate gestational-age specific Z-scores in US populations have not been published. Therefore, based on fit with our data, we selected reference populations from England and the Netherlands for our study. Although these reference populations appeared to closely resemble our study population we cannot rule out potential misclassification. Third, in 90 out of 223 mothers in our study, gestational age was estimated by the ultrasound examination itself. Although this could have affected the associations we observed, in our sensitivity analysis restricted to mothers with known LMP, our finding of an inverse association between HC and in utero exposure changed only slightly. Fourth, as our study examined associations between low levels of arsenic and fetal growth a considerable number of arsenic species in the maternal urine were below the limit of detection requiring assignment of values. Considering the high number of samples below the limit of detection particularly for arsenate and arsenite, this could have misclassified the total arsenic exposure at very low levels thus driving observed associations towards the null.

Lastly, our study is a cross-sectional analysis with fetal biometric measurements taken between 18 and 22 weeks gestation and in utero arsenic exposure taken between approximately 24 and 28 weeks of gestation. Therefore our study is not designed to evaluate a true cause-effect relationship; however, we suspect reverse causality to be unlikely (i.e., that reduced growth causes higher arsenic exposure). Further, the chronological gap in time between fetal growth and estimation of in utero arsenic exposure (by maternal urinary arsenic concentration which is a measure of recent exposure) could have contributed to misclassification. Nevertheless, there is evidence that urinary arsenic concentration is a fairly consistent estimate of total arsenic exposure over long periods of time [53, 54].

Despite these limitations, our study offers two important contributions to the literature. First, we have demonstrated that automated techniques can be used to extract biometric measurements successfully from EMRs. Second, our preliminary analyses offer important insights into the potential relationship between exposure to relatively low levels of arsenic and fetal growth in US populations. Future work is required to establish more conclusive findings and further evaluate the potential health effects related to in utero arsenic exposure.

## Electronic supplementary material

Additional file 1: Figure S1: Estimated fetal weight (A), head circumference (B), biparietal diameter (C), abdominal circumference (D), and femur length (E) across week of gestation (18 to 40 weeks) compared to reference population. In all panels, solid black line represents the mean of the reference population and dashed black lines represent the 5^th^ and 95^th^ percentiles of the reference population. (PDF 310 KB)

## Abbreviations

AC: Abdominal circumference
BMI: Body mass index
BPD: Biparietal diameter
CPT: Current procedural terminology
DMA: Dimethylarsinic acid
EFW: Estimated fetal weight
EMRs: Electronic medical records
FL: Femur length
HC: Head circumference
HPLC: High performance liquid chromatography
ICPMS: Inductively coupled plasma mass spectrometry
LMP: Last menstrual period
MMA: Monomethylarsonic acid
NHBCS: New Hampshire Birth Cohort Study.
