# Supplementary figures and images for: Preliminary analysis of in utero low-level arsenic exposure and fetal growth using biometric measurements extracted from fetal ultrasound reports
Source: Environ Health. 2015 Mar 30;14:12. doi: 10.1186/1476-069X-14-12 (PMC4429981; doi:10.1186/1476-069X-14-12)

Supplemental Material, Figure S1.

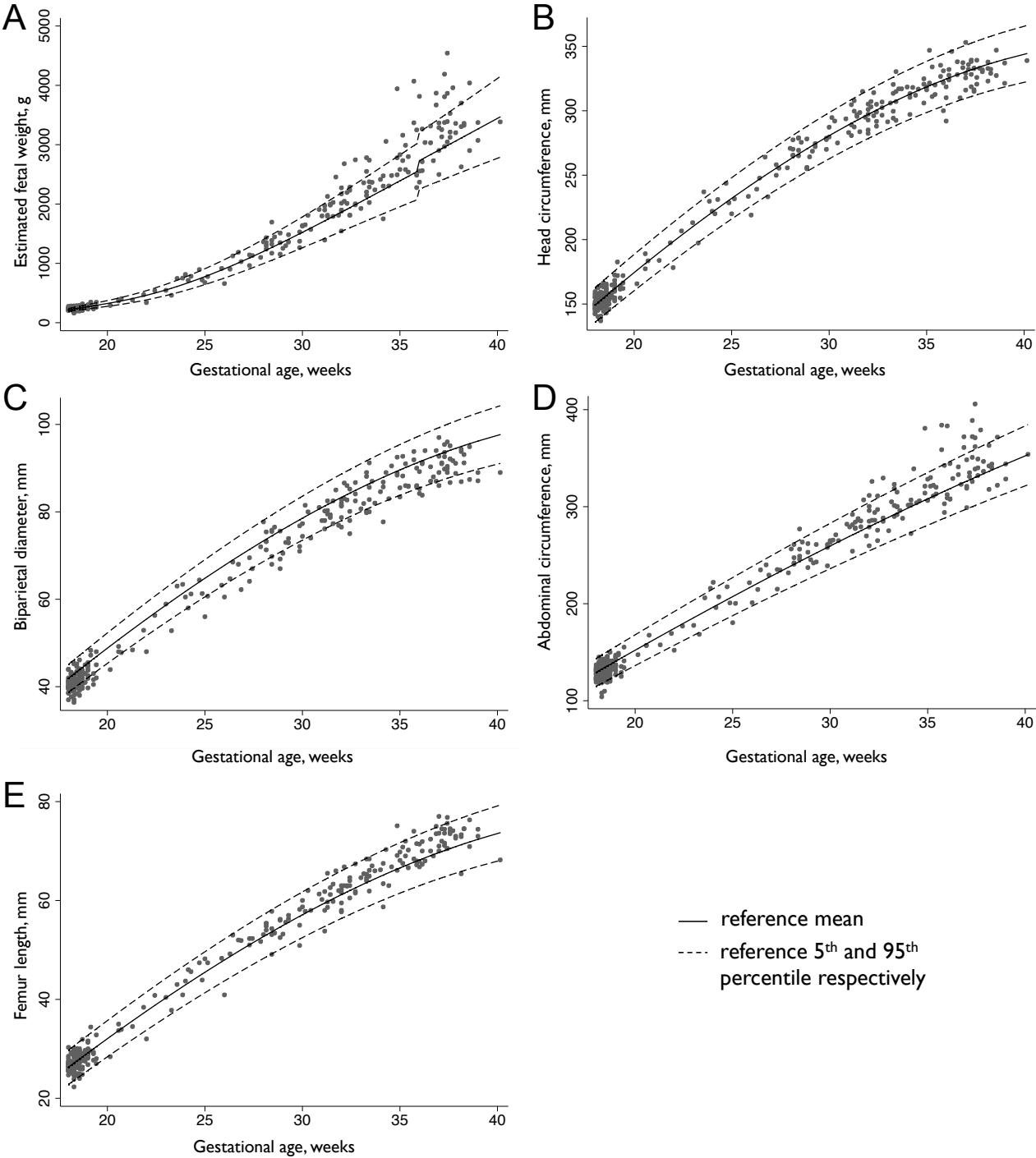

Supplement: Supplementary file 1 — Additional file 1: Figure S1: Estimated fetal weight (A), head circumference (B), biparietal diameter (C), abdominal circumference (D), and femur length (E) across week of gestation (18 to 40 weeks) compared to reference population. In all panels, solid black line represents the mean of the reference population and dashed black lines represent the 5th and 95th percentiles of the reference population. (PDF 310 KB) [file 12940_2014_850_MOESM1_ESM.pdf]
